# Neurotheology: The relationship between brain and religion

**Published:** 2014

**Authors:** Alireza Sayadmansour

**Affiliations:** Department of Philosophy, School of Literature and Humanities, University of Tehran, Tehran, Iran

**Keywords:** Brain, Causality, Functional Brain Imaging, Neurotheology, Prayer, Spirituality

## Abstract

“Neurotheology” refers to the multidisciplinary field of scholarship that seeks to understand the relationship between the human brain and religion. In its initial development, neurotheology has been conceived in very broad terms relating to the intersection between religion and brain sciences in general. The author's main objective is to introduce neurotheology in general and provides a basis for more detailed scholarship from experts in theology, as well as in neuroscience and medicine.

## Introduction

Neurotheology, also known as “spiritual neuroscience”^1^, is an emerging field of study that seeks to understand the relationship between the brain science and religion.^2^ Scholars in this field, strive up front to explain the neurological ground for spiritual experiences such as “the perception that time, fear or self-consciousness have dissolved; spiritual awe; oneness with the universe.”^3^ There has been a recent considerable interest in neurotheology worldwide. Neurotheology is multidisciplinary in nature and includes the fields of theology, religious studies, religious experience and practice, philosophy, cognitive science, neuroscience, psychology, and anthropology. Each of these fields may contribute to neurotheology and conversely, neurotheology may ultimately contribute in return to each of these fields. Ultimately, neurotheology must be considered as a multidisciplinary study that requires substantial integration of divergent fields, particularly neuroscience and religious phenomena. More importantly, for neurotheology to be a viable field that contributes to human knowledge, it must be able to find its intersection with specific religious traditions.^4^ For instance, Islam is powerful, growing religion that would seem to be an appropriate focus of neurotheology. After all, if neurotheology is unable to intersect with Islam, then it will lack utility in its overall goal of understanding the relationship between the brain and religion. Obviously, these resulting changes of behavior would lead to a better understanding or perception of the world around us creating better harmonious, functional individuals, who can be the driving force behind change and on a larger scale of family and society as well.

Clearly, one of the initial problems with neurotheology as a field is the exploitation of “neurotheology” as a term. Too often, the term “neurotheology” has been used inaccurately or inappropriately.^5^ Many times, it appears to refer to a study an idea that incorporates neither neuroscience nor theology. Strictly speaking, neurotheology refers to the field of scholarship linking the broad categories of both neurosciences and theological studies. Neuroscience would thus refer to the empirical study of the central nervous system or ‘brain and theology’; would refer to the critical and rational analysis of a particular religious belief system, pertaining to God. It may also constitute a robust “natural theology of the brain”.^6^ Of course, both the terms “neuroscience” and “theology” have evolved over time.

Neuroscience used to imply the study of nerve cells and their function without a clear regard for behavioral and cognitive correlates. Neuroscience today extends over many different fields including cognitive neuroscience, neurology, neurobiology of spirituality, psychiatry and psychology, and sociology. The tools have also become much more advanced, including a variety of brain imaging capabilities, for exploring the relationship between the brain and various cognitive, emotional, and behavioral processes (i.e. “photographs of God”^7^). Theology has also changed over time. In a very strict sense, theology is the study of God. Thus, the word “theology” should be reserved for theistic religions only, and even more specifically, from, those arising out of the Abrahamic traditions. Both Judaeo-Christian and Islamic theologians are divided among themselves about whether to integrate philosophy into their theologies, or to base their theologies solely upon their scriptures.

For neurotheology to be a viable field, it most likely should not be limited to only neuroscience and theology. In reconsidering the term “neurotheology” then, it seems appropriate to allow for expanded uses of the “neuro” component and the “theology” component. It would seem appropriate for neurotheology to refer to the totality of religion and religious experience as well as theology. This ability to consider, in a broad scope, all of the components of religion from a neuroscientific perspective would provide neurotheology with an abundant diversity of issues and topics that can ultimately be linked, under one heading. On the other hand, if the target of study encompasses so many aspects of religion and spirituality, the field might become so broad that it loses its ability to say something unique about religious and spiritual phenomena.

### Brain functions and theological topics

In this section, I will examine a variety of overarching brain functions to determine how theological concepts, in general, might be derived. It should be emphasized that the brain functions described below relate to broad categories of functions. Future neurotheological scholarship will need to better evaluate the specifics of different brain processes to determine if and how they relate to religious and theological concepts in general.^8^

### The holistic function

The brain, especially the right hemisphere, has the ability to perceive holistic concepts such that we perceive and understand wholeness in things rather than particular details. For example, we might understand all the cells and organs to comprise the whole human body. From a religious or spiritual perspective, we might understand a concept of absolute oneness as pertaining to God. Furthermore, the holistic process in the brain allows for the expansion of any religious belief or doctrine to apply to the totality of reality, including other people, other cultures, animals, and even other planets and galaxies. In fact, as human knowledge of the extent of the universe has expanded, the notion of God has incorporated this expanding sense of the totality of the universe. The holistic function pushes us to contemplate that whatever new reaches of the universe astronomers can find, God must be there. No matter how small and unpredictable a subatomic particle might be, God must be there, too.^9^

### The quantitative function

In the most general sense, the quantitative processes of the brain help to produce mathematics and a variety of quantitative-like comparisons about objects in the world. The quantitative function clearly both underlies and supports much of science and the scientific method.^10^ Science essentially is based upon a mathematical description of the universe. In terms of philosophical and theological implications, the quantitative function appears to have heavily influenced the ideas of philosophers such as Pythagoras who often used mathematical concepts such as geometry to help explain the nature of God and the universe.

A potentially interesting application of the quantitative function is in the evaluation of the strong emphasis on certain special numbers used in religious traditions. For example, specific numbers abound in the bible such as the number 40 (40 days and nights of the flood; the Jews wandered the desert for 40 years, etc.) and lend their significance in terms of time, people, and places. Islam also makes use of special numbers in the Quran and in the doctrines that follow from it. According to Shi'ism, there are the Ten Ancillaries of the Faith (Sunnis believe in the Five Pillars of Islam and the Six Articles of Belief); and the 99 attributes of Allah. One might ask if these numbers provide additional meaning within our brain. Is it easier for us to believe in or comprehend these concepts when presented along with a specific number? We know that our brain has a great interest in numbers and generally likes to utilize them. This quantitative process might strengthen our belief in anything associated with numbers. And again, there are special numbers such as 5, 10, 40, or 99, which might strike a particular effect in the brain, a function of the left hemisphere.

### The binary function

The binary processes of the brain enable us to set apart two opposing concepts. This ability is critical for theology since the opposites that can be set apart include those of good and evil, justice and injustice, and man and God among many more.^11^ Many of these polarities/dichotomies are encountered throughout religious texts of all religions. Much of the purpose of religions is to solve the psychological and existential problems created by these opposites. Theology, then, must evaluate the myth structures and determine where the opposites are and how well the problems presented by these opposites are solved by the doctrines of a particular religion such as Islam. The instructive styles of the Quran are often examples juxtaposing the good and the bad.

### The causal function

The ability of the brain to perceive causality is also crucial to theology. When the causal processes of the brain are applied to all of reality, it forces the question of what is the ultimate cause of all things.^12^ This eventually leads to the classic notion of “St. Thomas Aquinas's Uncaused First Cause” as an argument for God's existence. For monotheistic religions, the foundational doctrines posit that God is the uncaused cause of all things. However, this very question of how something can be uncaused is a most perplexing problem for human thought. In fact, theologians, philosophers, and scientists have tangled with causality as integral to understanding the universe and God. Aristotelian philosophy postulated four aspects of causality i.e. efficient causality, material causality, formal causality, and final causality****. The question of causality thus became applied to God to determine how, in fact, God could cause the universe.^13^

### Willfulness and orienting functions

Two other important brain functions are related to the ability to support willful or purposeful behaviors and the ability to orient our self within the world. Neuroscientifically, the willful function is regarded to arise, in large part, from the frontal lobes.^14^ There is evidence that frontal lobe activity is involved in executive functions such as planning, coordinating movement and behavior, initiating and producing language. Evidence has also shown the frontal lobes to become activated when an individual performs a meditation or prayer practice in which there is intense concentration on the particular practice.^15^

### Reflections on neurotheology and neuroscience

There are a number of neuroscience topics that might directly influence and be influenced by neurotheological research. One of the major issues that neurotheology faces, is the problem of the ability to determine the subjective state of the subject. This is also a more universal issue in the context of cognitive neuroscience.^16^ After all, one can never know precisely what a research subject is thinking at the precise moment of imaging. If you have a subject solving a mathematical task, one does not know if the person's mind wandered during the task. You might be able to determine if they did the test correctly or incorrectly, but that in and of itself cannot determine why they were right or wrong. The issue of the subjective state of the individual is particularly problematic in neurotheology.^17^ When considering spiritual states, the ability to measure such states empirically while not disturbing such states is almost impossible. Hence, it is important to ascertain as much as possible what the person thinks they are experiencing. Neurotheology research can help better refine subjective measurements. Spiritual and religious states are perhaps the best described of all states and thus, can be an important starting point for advancing research in the measurement of subjective states.

Another area in which neurotheology could provide important scientific information is in understanding the link between spirituality and health.^18^ A growing number of studies have shown positive, and sometimes negative, effects on various components of mental and physical health.^19^ Such effects include an improvement in depression and anxiety, enhanced immune system, and reduced overall mortality associated with individuals who are more religious. On the other hand, research has also shown that those individuals engaged in religious struggle, or who have a negative view of God or religion, can experience increased stress, anxiety, and health problems.^20^ Research into the brain's responses to positive and negative influences of religion might be of great value in furthering our understanding of the relationship between spirituality and health.

Finally, one of the most important goals of cognitive neuroscience is to better understand how human beings think about and interact with our environment. In particular, this relates to our perception and response to the external reality that the brain continuously presents to our deep consciousness.^21^ Neurotheology is in the unique position to be able to explore epistemological questions that arise from neuroscience and theology. Thus, integrating religious and scientific perspectives might provide the foundation upon which scholars from a variety of disciplines can address some of the greatest questions facing humanity.

## Conclusion

As an emerging field of study, neurotheology has the potential to offer a great deal to our understanding of the human mind, consciousness, scientific discovery, spiritual experience, and theological discourse. In particular, there are many potentially rich areas to consider in the context of Islam. It should be remembered that neurotheological scholarship must tread carefully upon these topics and attempt to develop clear, yet novel methods of inquiry. All results of neurotheological scholarship must be viewed and interpreted cautiously and within the context of existing doctrine, beliefs, and theology. However, if neurotheology is ultimately successful in its goals, its integrative approach has the potential to revolutionize our understanding of the universe and our place within it. Better understanding of human mind, its biology and neurocircuitry has the potential to solve man-made problems. It can even create a bridge between the empirical science of neurology with the intangibility and sensitivities of theology.
